# Incremental costs of hospital-acquired complications in Alberta, Canada

**DOI:** 10.1186/1472-6963-11-S1-A15

**Published:** 2011-10-19

**Authors:** T Jackson, A Fong, M Liu, K Murray, L Walz, C Houston, K Walker, S Dean

**Affiliations:** 1 Division of Pulmonary Medicine, Faculty of Medicine and Dentistry, University of Alberta, Edmonton, T6G 2C8, Canada; 2 Health Policy and Management, School of Public Health, University of Alberta, Edmonton, T6G 2C8, Canada; 3 Data Integration Measurement and Reporting, Strategy and Performance, Alberta Health Services, Calgary, Canada; 4 Provincial Operations, Standards and Strategies, Health Information, Alberta Health Services, Calgary, Canada

## Background

Hospital-acquired diagnoses (HAD) not only lengthen inpatients’ recovery times but also incur significant additional costs of care. The focus of previous research has been on ‘highly preventable’ indicator conditions and the cost of individual episodes, rather than on the entire spectrum of unintentional patient harm. As well, little attention has been paid to the frequency of HADs and resulting total costs.

## Objective

The objective of this study was to estimate the incremental cost, aggregated system costs, and length-of-stay effects of hospital-acquired diagnoses in eight Alberta (Canada) hospitals.

## Methods

Routinely coded diagnosis data, combined with a Present-on-Admission (POA) flag, were used to group 206,011 inpatient records into the 144 classes of the Classification of Hospital Acquired Diagnoses (CHADx). In Alberta’s larger hospitals, costs are measured using sophisticated bottom-up, patient-level costing systems.

We employed a generalized linear model (GLM) with a gamma distribution using a log link relationship between the total cost of hospitalization and all 144 CHADx groups, after controlling for in-hospital death, one-day hospitalization, and the mean of uncomplicated cases in each CaseMix Group (CMG).

## Results

Nearly a quarter of the sample (23.9%) had at least one recorded hospital-acquired diagnosis. Across all cases, any HAD was associated with increased costs of C$10,866, more than double the mean cost of an uncomplicated admission, with a mean of 4.7 additional days of stay. CHADx representing the highest per-episode median incremental cost included multi-drug resistant *Staph aureus* (CHADx 4.3, C$11,357), falls with fractured neck of femur (CHADx 3.1, C$6,679), and pressure ulcers (CHADx 8.1, C$6,512). Twenty-two CHADx added > 2 days to the median for a similar but uncomplicated stay.

Taking the volume of cases into account, and using an approximation of the mean incremental cost to capture all system costs, urinary tract infection (CHADx 9.2) was the most costly, adding C$19.3 million to system costs. CHADx responsible for the greatest extension of LOS (length of stay) across the system were similar to those adding the greatest costs, with the notable additions of *Clostridium difficile* infection (CHADx 7.3, +8,813 days) and septicaemia (CHADx 4.1, +6,284).

High-level grouping of CHADx showed hospital-acquired infections to be the mostly costly type of complication, adding C$49.6 million, although this finding is sensitive to the way in which HAD conditions are grouped.

## Discussion

Few hospital-acquired diagnoses are preventable in every case, but most have been shown to be amenable to a reduction in their rates. POA flags on routine diagnosis data considerably improve the ability to identify compromised patient care, although such data will remain controversial without further efforts to improve medical record documentation. Adding financial and length-of-stay dimensions to discussions about hospital quality improvement may strengthen efforts to reduce harm to patients, as will timely access to local data.

